# Novel Insights on Dietary Polyphenols for Prevention in Early-Life Origins of Hypertension: A Review Focusing on Preclinical Animal Models

**DOI:** 10.3390/ijms23126620

**Published:** 2022-06-14

**Authors:** You-Lin Tain, Chien-Ning Hsu

**Affiliations:** 1 Department of Pediatrics, Kaohsiung Chang Gung Memorial Hospital and Chang Gung University College of Medicine, Kaohsiung 833, Taiwan; tainyl@cgmh.org.tw; 2 Institute for Translational Research in Biomedicine, Kaohsiung Chang Gung Memorial Hospital, Kaohsiung 833, Taiwan; 3 Department of Pharmacy, Kaohsiung Chang Gung Memorial Hospital, Kaohsiung 833, Taiwan; 4 School of Pharmacy, Kaohsiung Medical University, Kaohsiung 807, Taiwan

**Keywords:** polyphenols, hypertension, resveratrol, developmental origins of health and disease (DOHaD), gut microbiota, oxidative stress, nitric oxide

## Abstract

Polyphenols are the largest group of phytochemicals with health benefits. Early life appears to offer a critical window of opportunity for launching interventions focused on preventing hypertension, as increasing evidence supports the supposition that hypertension can originate in early life. Although polyphenols have antihypertensive actions, knowledge of the potential beneficial action of the early use of polyphenols to avert the development of hypertension is limited. Thus, in this review, we first provide a brief summary of the chemistry and biological function of polyphenols. Then, we present the current epidemiological and experimental evidence supporting the early-life origins of hypertension. We also document animal data on the use of specific polyphenols as an early-life intervention to protect offspring against hypertension in adulthood and discuss underlying mechanisms. Continued research into the use of polyphenols to prevent hypertension from starting early in life will have far-reaching implications for future health.

## 1. Introduction

Polyphenols are the largest group of phytochemicals, all of which are natural compounds synthesized entirely by plants [1]. Polyphenols are generally categorized as flavonoids and nonflavonoids. Flavonoids have a chemical structure of 15 carbons constituted by a common skeleton with a C6-C3-C6 structure. Polyphenols are potent antioxidants and have been linked to many health benefits [2,3,4]. Polyphenols display a large range of biological effects, including antioxidant properties, anti-inflammatory effects, anticancer activity, improvement of endothelial function, antiobesity activity, antidiabetic activity, antiatherosclerotic properties, restoration of NO bioavailability, etc. [2,3,4,5]. However, further trials are required to provide recommendations on the dietary reference intake of polyphenols for health and disease prevention, and to fully assess the molecular mechanisms of action [5].

Increasing evidence has demonstrated the beneficial role of polyphenols in the treatment of hypertension [6,7,8]. Hypertension is one of the most important risk factors for cardiovascular disease (CVD), which is the primary cause of mortality worldwide [9]. The WHO estimates that more than a billion people have hypertension, upwards of 1 in 4 men and 1 in 5 women [10]. Even though pharmacological and interventional strategies have advanced in the past decades, the worldwide prevalence of hypertension is still high and continues to grow [11]. As the scope of the condition expands, greater attention should be focused on preventing and not just treating hypertension.

Although hypertension is an inheritable condition, genetic variants explain only a tiny fraction of phenotypic variations and disease risk [12]. Prior work suggested that missing heritability in hypertension can be a result of adverse events during prenatal, perinatal, or early postnatal life. Indeed, growing evidence supports the supposition that hypertension can originate in early life, resulting from a complex interplay of genetic, epigenetic, and environmental factors [13,14,15].

The link between one’s environment in early life and disease as an adult is summed up in the concept of developmental origins of health and disease (DOHaD) [16]. Particularly, adverse programming processes can be avoided or postponed by early intervention, that is, through reprogramming, to avoid the development of chronic diseases across the lifespan [14,17]. A broad spectrum of environmental stimuli can induce the early-life origins of hypertension, including maternal malnutrition, illness, substance abuse, toxin/chemical exposure, medication use during pregnancy, etc. [14,15,18,19,20,21,22].

During pregnancy and lactation, a plant-based diet can effectively meet energy and nutrient needs [23]. It is known that plant-based diets are rich in polyphenols; however, the protective or deleterious effects of polyphenol-rich foods on chronic diseases in pregnant women remain unclear [24]. Published data support the idea that early-life treatment with certain polyphenols can counteract the adverse processes behind developmental programming and thereby prevent the development of chronic diseases later in life [25]. Although polyphenols have been shown to have benefits for hypertension, the literature focusing on maternal polyphenol supplementation to avoid the early-life origins of hypertension remains limited.

To attain the goal of this review, an electronic search of two bibliographic databases, Medline/PubMed and Embase, was performed without restricting the time of publication. The search keywords were as follows: “polyphenol”, “flavonoid”, “flavans”, “stilbenes”, “flavanones”, “isoflavones”, “anthocyanins”, “lignans”, “tannins”, “resveratrol”, “hypertension”, “blood pressure”, “pregnancy”, “mother”, “maternal”, “gestation”, “lactation”, “neonatal”, “perinatal”, “developmental programming”, “DOHaD”, “offspring”, “progeny”, and “reprogramming”. Relevant articles published in English-language journals were reviewed to find suitable reports. Additional studies were recruited based on references in eligible reports.

## 2. Polyphenol: Chemistry and Biological Function

The word “polyphenol” is a generic term derived from Greek: “poly” means many, and “phenol” is an aromatic ring with a hydroxyl group attached. Phenolic compounds are secondary metabolites broadly spread in the plant kingdom that can be categorized as flavonoids and nonflavonoids. So far, more than 8000 phenolic structures are known, and among them, around 5000 flavonoids have been discovered [2]. Phenolic compounds comprise one (phenolic acid) or more (polyphenol) aromatic rings with attached hydroxyl groups. Polyphenols are found in plant-based foods and beverages, notably fruits, vegetables, whole grains, chocolate, wine, and tea.

**Figure 1:**
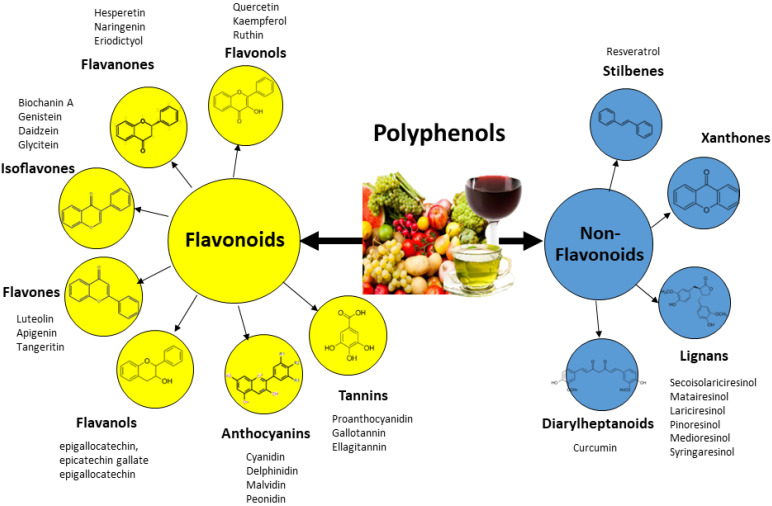
Polyphenol classes, chemical structures, and main compounds.

Polyphenols have been classified by their chemical structure, biological function, and source of origin [2]. In the interest of brevity, classification of polyphenols in this review is done based on the chemical structure. As illustrated in Figure 1, the flavonoids mainly present in foods are flavonols, flavanones, isoflavones, flavones, flavan-3-ols, and anthocyanins. Among the nonflavonoid phenolic compounds are xanthones, stilbenes, lignans, and tannins. Here, for the sake of brevity, we provide only a concise overview as an introduction to the chemistry of polyphenols. For more in-depth information, please refer to reviews published elsewhere [1,2]. 

### 2.1. Flavonoids

One of the most extensively studied groups of polyphenols is the flavonoids. Daily intake of flavonoids constitutes about two-thirds of the total intake of dietary polyphenols. Flavonoids have the C6–C3–C6 general structural backbone, in which the two C6 units are of phenolic nature. Flavonoids can be further classified into different subgroups based on the hydroxylation pattern and variations in the chromane ring, such as flavones, flavanones, isoflavones, flavanols, flavonols, and anthocyanins. 

A diverse range of pharmacological activities, including antioxidant, anti-inflammatory, antibacterial, antihyperlipidemic, and cardioprotective effects, are attributed to flavonoids [26]. Quercetin and kaempferol are the main representative flavonol molecules. Quercetin is mostly present in apples, onions, and berries, and has shown antihypertensive action [7]. Flavanones include naringenin, hesperetin, and eriodictyol. Flavanone intake has been linked to a reduced risk of obesity and diabetes [27]. 

The presence of isoflavones is almost entirely restricted to the leguminous family of plants. Isoflavones include biochanin A, genistein, daidzein, and glycitein [28]. The leading dietary source of isoflavones is soybean, which contains mainly genistein and daidzein. The chemical structure of isoflavones enables their attachment to and activation of estrogen receptors. Accordingly, isoflavones can exert estrogenic or antiestrogenic effects [28]. 

The basic chemical structure of flavones is in the form of two benzene rings united by a heterocyclic pyrone ring [29]. The main flavones in food are luteolin, apigenin, and tangeritin. Although flavones have demonstrated many potentially beneficial activities, they are not well absorbed compared to other polyphenols. 

Flavanols, or flavan-3-ols, are usually termed catechins [30]. The main sources of flavanols are cocoa, dark chocolate, and berries. Unlike most flavonoids, flavonols have no C4 carbonyl in ring C and no double bond between C2 and C3. They can also form gallic acid conjugates such as epigallocatechin, epicatechin gallate, and epigallocatechin gallate [30]. Cocoa and chocolate are rich in flavonols, which has attracted attention as an option for the prevention of CVD and hypertension [31]. 

Represented by over 600 structures identified to date, anthocyanins are naturally occurring plant pigments [32]. Specifically, cyanidin, delphinidin, malvidin, and pelargonidin are widely distributed in plants [33]. Similar to other flavonoids, anthocyanins also have a number of health benefits [32,33]. 

Tannins are water-soluble, high-molecular-weight polyphenolic compounds that are categorized into two major groups: hydrolyzable and nonhydrolyzable. Hydrolyzable tannins are further classified into gallotannins and ellagitannins. Proanthocyanidins, better known as condensed tannins, are flavonoid polymers that exist widely in common foods [34]. Tannins provide protection against a broad range of biotic and abiotic stressors and have several pharmacological effects involving antihypertension [34,35].

### 2.2. Nonflavonoids

As shown in Figure 1, nonflavonoids phenolic compounds include xanthones, stilbenes, lignans, and diarylheptanoids [1,2]. Xanthones comprise a family of O-heterocycle symmetrical compounds with a dibenzo-γ-pyrone scaffold. Their distinctive tricyclic aromatic ring gives them cardioprotective potential and a broad spectrum of physiological properties [36]. Stilbenes are a small family of phenylpropanoids produced in a number of plant species. The basic chemical structure of stilbenes consists of a C6-C2-C6 skeleton, usually with two isomeric forms. [37]. Resveratrol, from grapes and red wine, is one of the best-studied stilbenes [38]. 

Lignans form a group of phenolic compounds with a backbone of two phenylpropanoid (C6-C3) units [39]. Plant lignans occur in the form of glycosides. Compared to other phenolic compounds, lignans are relatively less studied even though they are widely distributed. 

Diarylheptanoids are phenolic compounds with a skeletal structure of two aromatic rings conjugated with seven carbon chains [40]. Diarylheptanoids have been used as nutraceuticals due to their broad array of health-promoting properties [41]. Among nutraceuticals, curcumin is an important diarylheptanoid compound, which has been studied widely for its role in protection against many diseases [42]. The antihypertensive effect of curcumin has been reported in spontaneously hypertensive rats [43].

### 2.3. Biotransformation and Bioavailability of Polyphenols

**Figure 2:**
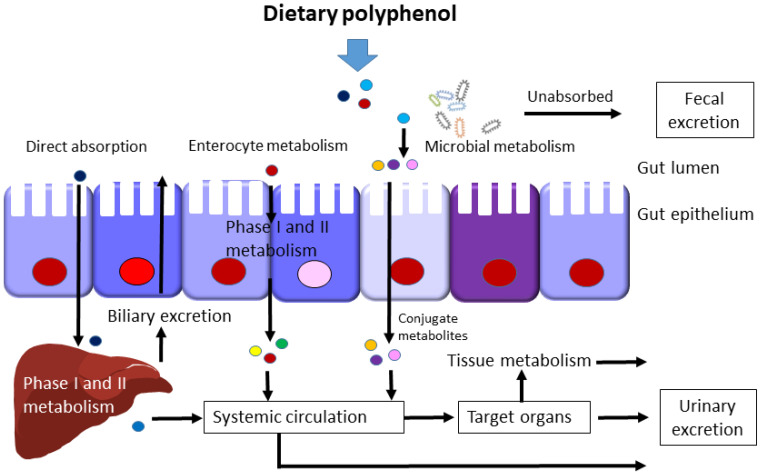
Metabolic fate of dietary polyphenols in the body. Within the host, dietary polyphenols undergo phase I and II metabolism in liver and gut, microbial metabolism, absorption in systemic circulation, interaction with target organs, and elimination in feces and urine.

The metabolic fate of dietary polyphenols in the body is schematically displayed in Figure 2. Only a minor portion of dietary polyphenols (5–10% of total polyphenol intake) can be directly absorbed in the small intestine, generally after deconjugation reactions such as deglycosylation [44]. After absorption, these less complex polyphenolic compounds undergo phase I and II reactions in the liver and enterocytes, giving rise to a series of water-soluble conjugate metabolites that are rapidly released into the systemic circulation for further organ distribution and urinary excretion. The remaining unabsorbed polyphenols (90–95% of total polyphenol intake) are known to be metabolized by gut microbes. 

The biological characteristics of polyphenols are determined by intestinal absorption and bioavailability. Bioaccessibility, which determines the release and solubility of bioactive compounds during digestion for further absorption, is a crucial factor in bioavailability. Most polyphenolic compounds show low bioavailability, which is mainly linked to their poor bioaccessibility [45]. Importantly, gut-microbiota-derived metabolism and intestinal absorption affect the bioaccessibility of polyphenols [46].

The gut microbiota is responsible for the extensive degradation of the original polyphenolic structures into multiple low-molecular-weight phenolic metabolites. Polyphenol metabolites have attracted great interest, as many of them have shown similar biological effects compared to the parent compounds. There is a two-way mutual reaction between polyphenolic compounds and the gut microbiota that has an impact on human health. First, the gut microbiota mediates the biotransformation of polyphenols into their microbial metabolites, helping to increase their bioavailability. Second, polyphenols can act as prebiotics to shape gut microbiota composition and enhance beneficial bacteria [47]. 

As an example, the catabolic transformation of resveratrol has been extensively studied in recent years. In humans, resveratrol is mainly absorbed orally (approximately 70%) [47]. Resveratrol absorption occurs by diffusion or by forming complexes with membrane transporters. In the liver, sulfation and glucuronidation are the principal phase II metabolic pathways of resveratrol. As a result, the free form of resveratrol is at very low levels in the circulation [48]. In the circulation and target organs, the major forms of resveratrol are sulfate (e.g., trans-resveratrol-3-sulfate) and glucuronide (e.g., trans-resveratrol-3-glucoronide) conjugate metabolites. Other resveratrol derivatives, such as dihydroresveratrol and piceatannol, are also detectable in target organs [49,50]. Once metabolized, resveratrol can be rapidly excreted, with an elimination half-life of 130–180 min [47].

In addition, the gut microbiota is involved in resveratrol catabolism by increasing its availability from resveratrol precursors and producing resveratrol derivatives [49]. Showing high inter-individual variation, absorption of orally ingested resveratrol in humans and rats has been reported at approximately 20–70% and 15–50%, respectively [51,52]. These data indicate that the bioavailability of resveratrol largely differs from one person to another, depending mainly on the administration rate and dose, as well as the gut microbial environment.

### 2.4. Beneficial Effects of Polyphenols in Hypertension

Many polyphenol-containing foods and beverages, such as grapes, tea, cocoa, and soy products, have been studied as antihypertensive agents [6]. The basic chemical aspects of flavonols, flavanols, isoflavones, anthocyanins, and stilbenes, as agents possibly responsible for the observed effects of polyphenol-rich foods on BP, are addressed. The reported mechanisms mediating the protective effects of polyphenols in hypertension, mainly supported by experimental data in animals, include inhibition of oxidative stress, enhancement of nitric oxide (NO) bioavailability, improvement of endothelial function, inhibition of vasoconstrictor endothelin-1 synthesis, and regulation of the renin–angiotensin–aldosterone system (RAAS) [6,7,53,54]. 

Although several systematic reviews indicated that dietary flavonoid intake reduces CVD risk [39,55,56], some data did not suggest that flavonoid-rich fruits can affect systolic and diastolic BP [57]. In addition, one meta-analysis that examined 45,732 cases of hypertension from 20 studies demonstrated that flavonoid intake showed a nonsignificant association with decreased risk of hypertension, while dietary anthocyanin intake was associated with an 8% reduction in hypertension risk [58]. Even when the data are inconclusive and many questions remain open, on the whole, the evidence is encouraging to start considering polyphenol intake that can provide benefits to hypertensive subjects. 

## 3. Early-Life Origins of Hypertension

### 3.1. Epidemiological Evidence 

There are several lines of evidence to support the idea that early-life environmental stimuli are closely linked to the risk of hypertension later in life. The first is observations from famine. Children born to women exposed to famine develop multiple chronic diseases involving hypertension in later life [59,60,61]. Another line of evidence comes from mother–child cohorts. Prior work found several risk factors related to the early-life origins of hypertension, including maternal malnutrition [62], maternal obesity [63], gestational hypertension [64], short-term breastfeeding [65], low maternal vitamin D levels [66], maternal smoking [67], and environmental chemical exposure [22].

The third line is many studies indicating that preterm birth and low birth weight (LBW) are key risk factors for hypertension later in life [13,68,69,70]. A meta-analysis study of 1342 preterm babies reported that preterm or very LBW babies had higher systolic BP in adulthood [70]. Further, in twin studies, associations have been reported between LBW and hypertension [71,72,73]. 

However, such epidemiological studies are unable to test direct cause-and-effect relationships or provide the molecular mechanisms that underlie the programming processes in order to develop efficient early-life interventions. Hence, animal models have been created to establish the biological plausibility of the associations observed in epidemiological studies, providing proof of causality.

### 3.2. Experimental Evidence 

A wide range of early-life insults using animal models to study the early-life origins of hypertension has been reported, including maternal malnutrition, maternal illness, pregnancy complications, environmental chemical exposure, and medication use in pregnancy [14,15,18,19]. Several small (e.g., rat and mouse) and large (e.g., ewe and cow) animal models have been used to assess the early-life origins of hypertension, with rats the most commonly used species [15,74,75,76]. So far, animal models have provided significant insights into the pathophysiological mechanisms involved in the early-life origins of hypertension. These molecular mechanisms include but are not limited to oxidative stress [20], dysregulated NO signaling [77], aberrant activation of the RAAS [78], dysfunctional nutrient-sensing pathways [79], dysbiotic gut microbiota [80], and epigenetic regulation [81]. As detailed descriptions of these mechanisms are beyond the scope of this review, readers are referred to reviews elsewhere for more in-depth information. 

**Figure 3:**
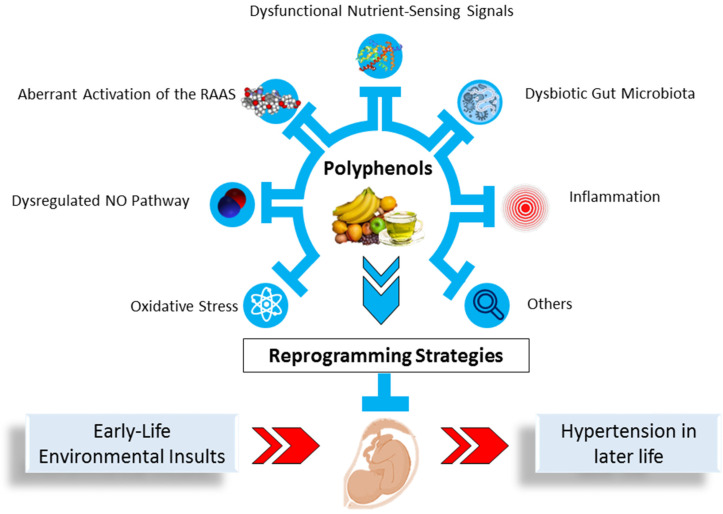
Schema outlining protective role of polyphenols as a reprogramming strategy for mediating common mechanisms behind early-life origins of hypertension programmed by environmental insults. NO, nitric oxide; RAAS, renin–angiotensin–aldosterone system.

While the mechanisms underlying the early-life origins of hypertension remain to be fully elucidated, our knowledge of potential molecular mechanisms has advanced in recent years by running experiments on animals, which aid in developing efficient early intervention measures, specifically reprogramming, to prevent hypertension from happening [14,17]. Given that polyphenols regulate many biological functions, we might presume that using them as an early-life intervention could reprogram adverse programming processes and prevent the development of hypertension throughout life. A summary of the links between polyphenols and protective mechanisms implicated in the early-life origins of hypertension is given in Figure 3.

## 4. Polyphenols as a Reprogramming Strategy

So far, no information is available from human clinical studies with regard to the effects of perinatal polyphenol supplementation on the offspring’s BP. Given that polyphenols appear to offer many promising health benefits, and that many polyphenols are claimed as nutraceuticals, it is no wonder supplementation with polyphenols during gestation and/or lactation has been examined in animal models to improve maternal and fetal outcomes [24,82,83,84]. 

Among the animal studies that analyzed polyphenol compounds in the context of DOHaD-related disorders, many focused on the impact of resveratrol. Our understanding of the potential beneficial effects of early polyphenol supplementation to prevent hypertension of developmental origins is limited. Thus, in this review, we summarize experimental evidence documenting the use of polyphenols to prevent hypertension considering early-life interventions through pregnancy and lactation, which is presented in Table 1 [85,86,87,88,89,90,91,92,93,94,95,96,97]. The studies are limited to those that evaluated offspring outcomes starting post-weaning.

In the current review, rats were found to be the most widely used animal species. Only a mouse model was reported with regard to the protective effects of quercetin treatment against maternal high-fat diet-induced hypertension [85]. The reprogramming effects of polyphenol supplementation in rats range from 12 weeks to 6 months of age, which equates to human ages from adolescence to adulthood [98], while there is a paucity of information on the long-term effects of early polyphenol intervention on offspring in old age.

Numerous rat models of early-life insults, such as high-fat diet [85,87,89,95,97], antenatal dexamethasone exposure [86], maternal chronic kidney disease (CKD) [88], N^G^-nitro-L-arginine-methyl ester (L-NAME) administration [90], prenatal asymmetric dimethylarginine (ADMA) and trimethylamine N-oxide (TMAO) exposure [91], prenatal 2,3,7,8-tetrachlorodibenzo-p-dioxin (TCDD) exposure [92], prenatal TCDD plus dexamethasone exposure [93], maternal prenatal bisphenol A and high-fat diet exposure [94], and maternal hypertension [96], have been established to evaluate the reprogramming effects of various polyphenols on hypertension in the offspring. As shown in Table 1, several groups of polyphenols, including flavonols [85], flavanols [86,87], stilbenes [88,89,90,91,92,93,94,95,96], and tannins [97], have been evaluated in animal models of the early-life origins of hypertension. Among them, resveratrol is the most common polyphenol being studied [88,89,90,91,92,93,94,95,96]. In some studies, the types and amounts of active phenols administered to animals were not reported in detail [87,97]. One study used garlic oil, since garlic contains important biologically active compounds involving polyphenols. The flavonoids in black garlic are mainly catechin, epigallocatechin, and epigallocatechin gallate [99]. However, different garlic cultivars have varied flavonoid and phenolic contents, which differentially determines their health properties [100]. In addition to phenolic compounds, garlic includes organosulfur substances and saponins [101]. Another study, using grape skin extract (ACH09), obtained about 30% of total polyphenols [97]. Given that the major active polyphenols were not determined in these studies, the extent of the protective effect of polyphenols deserves additional research.

A previous study revealed that a soy isoflavone-deficient diet during gestation results in elevated BP in male adult offspring, which can be prevented by switching to a soy isoflavone-rich diet for 6 months in adulthood [102]. Additionally, prior research has shown the antihypertensive effects of flavones, flavanones, anthocyanins, and xanthones [6,103,104]. Accordingly, whether these polyphenols also have their own protective effects in the early-life origins of hypertension should be determined by further research.

All of these observations provide insight into several core mechanisms behind the protective effects of polyphenols, including oxidative stress, dysregulated NO pathway, aberrant RAAS activation, dysfunctional nutrient-sensing signals, dysbiotic gut microbiota, and inflammation. The interconnection between polyphenols and the proposed protective mechanisms underlying hypertension programming in response to adverse early-life insults is depicted in Figure 3. These will be discussed in detail in the following sections.

## 5. Potential Core Mechanisms Reprogramming by Polyphenols

### 5.1. Oxidative Stress

One of the protective mechanisms of polyphenols and their metabolites is against oxidative stress [105]. The antioxidant activities of polyphenols are interrelated with their capacity to scavenge reactive oxygen species (ROS), upregulate antioxidant defenses, inhibit NADPH oxidase, increased glutathione (GSH) levels, and increase NO bioavailability [105,106].

Since the fetus has low antioxidant capacity, overproduction of ROS under suboptimal intrauterine conditions prevails over antioxidant defenses, giving rise to oxidative stress damage and consequently fetal programming [107]. As illustrated in Table 1, several early-life insults link oxidative stress to hypertension of developmental origins, including high-fat diet [85,95,97], antenatal dexamethasone exposure [86], maternal CKD [88], prenatal TCDD and dexamethasone exposure [93], and maternal bisphenol A and high-fat exposure [94].

As an antioxidant, quercetin has been used as a nutraceutical to offer protection against various diseases [106]. In a mouse model, adult offspring of dams fed a high-fat diet during pregnancy exhibited hypertension, which was protected by quercetin supplementation in the pregnant dam [85]. Another study revealed that maternal treatment with epigallocatechin gallate attenuated the developmental programming of hypertension induced by antenatal dexamethasone administration [86]. 

Resveratrol, a stilbene, has been widely explored in many diseases [36]. In a maternal CKD model, perinatal resveratrol supplementation protecting against hypertension was related to reduced expression of renal 8-hydroxy-2′-deoxyguanosine (8-OHdG, a biomarker for assessing oxidative DNA damage) [88]. Additionally, the effect of perinatal resveratrol therapy in reducing oxidative stress is evidenced by the protection against hypertension in adult progeny of dams exposed to TCDD and dexamethasone [93], and to bisphenol A and a high-fat diet [94]. Moreover, supplementation with grape skin tannins in pregnancy and lactation protects against hypertension induced by a maternal high-fat diet, accompanied by restoration of decreased superoxide dismutase, catalase, and glutathione peroxidase activity [97]. These observations indicate that the interplay between polyphenols and oxidative stress is implicated in the early-life origins of hypertension.

### 5.2. Dysregulated NO Pathway

NO, a potent vasodilator, plays a crucial role in pregnancy and fetal development. Ample evidence indicates that a dysregulated NO pathway contributes to the pathogenesis of hypertension developing in early life [77]. Asymmetric dimethylarginine (ADMA) is an NOS inhibitor [108]. As a reprogramming strategy, restoring the ADMA-related ROS/NO imbalance has been proposed to avert developmental programming and avoid the resulting hypertension [109]. Table 1 shows the reprogramming effects of polyphenols targeting the ADMA/NO pathway to avert hypertension of developmental origins reported in various animal models, including maternal high-fat diet [87,95], maternal CKD [88], maternal L-NAME administration [90], prenatal ADMA and TMAO exposure [91], prenatal 2,3,7,8-tetrachlorodibenzo-p-dioxin (TCDD) exposure [92], prenatal TCDD plus dexamethasone exposure [93], maternal prenatal bisphenol A and high-fat diet exposure [94], and maternal hypertension [96].

Garlic is a polyphenolic and organosulfur-enriched nutraceutical [110]. Garlic oil supplementation during gestation and lactation was reported to protect against maternal high-fat diet-induced hypertension in adult rat offspring, coinciding with decreased ADMA levels and increased NO bioavailability [87]. 

Prior research reveals that resveratrol can stimulate NO production via upregulating endothelial NOS expression, stimulating NOS activity, reducing oxidative stress, and reversing eNOS uncoupling [111]. Our previous study revealed that perinatal resveratrol supplementation reduced plasma ADMA levels and restored NO bioavailability, providing protection against hypertension in offspring programmed by a high-fat diet [95].

### 5.3. Aberrant Activation of the RAAS

The RAAS is a major hormone cascade involved in the regulation of BP [112]. It contains two opposite pathways: the classic angiotensin-converting enzyme (ACE)–angiotensin (Ang) II–angiotensin type 1 receptor (AT1R) pathway, mediated primarily by Ang II, and the nonclassic ACE2–angiotensin-(1-7)–Mas receptor axis, mediated mainly by angiotensin-(1-7). It is well known that aberrant activation of the classic RAAS leads to hypertension. Conversely, inhibition of the classic RAAS or activation of the nonclassic RAAS can prevent the development of hypertension [112].

In line with previous studies showing the antihypertensive actions of several polyphenols in hypertensive models [113,114,115], maternal resveratrol supplementation was shown to protect adult offspring against hypertension in rat models of prenatal ADMA and TMAO exposure [91], prenatal TCDD plus dexamethasone exposure [93], and a high-fat diet [95]. Hypertension in offspring programmed by a maternal high-fat diet was associated with increased Ang I levels and reduced Ang (1–7) levels in the plasma [95]. Resveratrol treatment reversed these changes but decreased plasma Ang II levels. Together, the RAAS signals affected by polyphenol resveratrol appear to be in favor of vasodilatation. Still, the detailed protective mechanisms behind the modulation of RAAS components by different polyphenols involved in the early-life origins of hypertension await further exploration.

### 5.4. Dysfunctional Nutrient-Sensing Signals

Nutrient-sensing signals have a decisive role in fetal development and are mainly determined by maternal nutrition [116]. Resveratrol has been well-studied for its role in regulating nutrient-sensing signals. Several signals, such as AMP-activated protein kinase (AMPK), sirtuin 1 (SIRT1), and peroxisome proliferator-activated receptor (PPARs), are molecular targets of resveratrol [117]. Resveratrol is an AMPK or SIRT-1 activator [118]. Given that AMPK and SIRT-1 can mediate the expression of PPAR target genes, and that several PPAR target genes contribute to the pathogenesis of hypertension [119], dysfunctional nutrient-sensing signals appear to be a core mechanism behind hypertension of developmental origins. On the contrary, the use of early-life interventions targeting AMPK signaling has been proposed to prevent the early-life origins of hypertension [120].

Supplementation with resveratrol during rat pregnancy and lactation protected against the rise in BP programmed by maternal L-NAME and a high-fat diet [90]. Sixteen-week-old offspring of dams treated with resveratrol presented activation of the AMPK/SIRT1 pathway. The same maternal intervention with resveratrol also showed beneficial effects against hypertension programmed by a high-fat diet coinciding with activation of nutrient-sensing signals [89]. These observations highlight the need to better elucidate preventive aspects concerning the interconnection between polyphenols and nutrient-sensing signals in early life implicated in hypertension of developmental origins.

### 5.5. Dysbiotic Gut Microbiota

Adverse maternal conditions can alter the offspring’s gut microbiota composition, resulting in adverse offspring outcomes [121]. Considering that polyphenols are biotransformed into their metabolites by gut bacteria and polyphenols can act like prebiotics to shape gut microbiota, it is speculated that maternal polyphenol supplementation has potential benefits in preventing hypertension of developmental origins. Indeed, flavanols and stilbenes have shown benefits in the early-life origins of hypertension in models of maternal high-fat diet, maternal CKD, and L-NAME plus high-fat diet [87,88,90]. 

In a high-fat diet model [87], maternal garlic oil therapy protected adult offspring against programmed hypertension associated with shifts in gut microbiota, with remarkable increases in the genera *Bifidobacterium* and *Lactobacillus*, two well-known probiotic strains. Additionally, garlic oil treatment increased plasma levels of acetate, propionate, and butyrate, which are the main microbiota-derived metabolites involved in BP control [122]. Given that the type and amount of active polyphenols were not determined in this study, the extent of the beneficial effect of garlic oil attributed to polyphenols deserves to be explored more fully.

Similarly, perinatal resveratrol supplementation protected against maternal CKD-induced hypertension in adult rat offspring, which was related to increased proportions of *Lactobacillus* and *Bifidobacterium*, as well as increased microbial richness and diversity [88]. In a maternal L-NAME plus high-fat diet model [90], the beneficial actions of resveratrol against hypertension of developmental origins are likely related to its ability to reduce the ratio of *Firmicutes* to *Bacteroidetes*, a microbial marker for hypertension [122]. It is an important proof of concept that polyphenols used early may act as prebiotics by reshaping the offspring’s gut microbiome and reprogramming the early-life origins of hypertension. 

Of note, the low bioavailability of polyphenols limits their clinical translation [45]. In this regard, we improved the efficacy of resveratrol via esterification to form resveratrol butyrate ester [123]. Our data show that low-dose resveratrol butyrate ester (25 mg/L) is as effective as resveratrol (50 mg/L) in preventing CKD-induced hypertension [124]. Considering that polyphenol bioavailability is mainly determined by gut microbiota, it would be important to further evaluate how gut microbiota affects polyphenol bioavailability involved in protecting against hypertension of developmental origins.

### 5.6. Inflammation

Pregnancy is considered to be a systemic physiologic inflammatory response, and inflammatory pathways are involved in compromised pregnancies and associated complications [125]. Polyphenols have been proposed to be useful as therapy for many diseases because of their anti-inflammatory actions [105]. Moreover, polyphenols can regulate immunity by interfering with immune cell regulation, gene expression, and proinflammatory cytokine synthesis [126].

The accumulation of T cells, macrophages, and their derived proinflammatory cytokines is involved in the pathogenesis of hypertension [127]. In addition, an imbalance of T helper 17 (TH17) and T regulatory (Treg) cells has been connected to hypertension [127]. The dysregulated Treg/TH17 balance and inflammation can be triggered via the aryl hydrocarbon receptor (AhR) signaling pathway [128]. The activation of AhR signaling can initiate inflammation by increasing monocyte adhesion, upregulating proinflammatory cytokine expression, inducing endothelial adhesion molecules, and reducing NO bioavailability [129]. 

A previous study showed that TCDD-induced hypertension coincided with TH17-induced renal inflammation, as well as AhR signaling activation [92]. Conversely, TCDD-induced activation of AhR signaling and TH17 responses can be restored by resveratrol supplementation during gestation and lactation. In addition, resveratrol was reported to act like an AhR antagonist, showing benefits in preventing offspring hypertension in other models of the early-life origins of hypertension [93,94]. 

Though the vast number of published studies proved the anti-inflammatory role of various types of polyphenols in prevention and therapy of many diseases [105], only resveratrol has been examined for its anti-inflammatory action in the early-life origins of hypertension. More work is required to gain a comprehensive insight into the role of polyphenols in modulating inflammatory cellular pathways in order to develop inflammation-targeted therapies for the prevention of hypertension of developmental origins.

### 5.7. Others

With regard to the multifaceted biological role of polyphenols, other possible mechanisms might be involved, for example, epigenetic regulation or regulation of H_2_S. Several polyphenols have epigenetic action [82]. Epigenetic deregulation has been identified as a molecular mechanism underlying developmental programming in the context of DOHaD [82]. Although one report showed that resveratrol therapy prevents obesity in adult progeny, accompanied by epigenetic regulation of leptin and its receptor through DNA methylation [130], the data are insufficient to conclude that the reprogramming effects of resveratrol on programmed hypertension are directly through epigenetic regulation. Additionally, several polyphenols have been reported to regulate H_2_S oxidation [131,132]. Notably, the protective effect of garlic oil on maternal high-fat diet-induced programmed hypertension is relevant to the enhanced H_2_S signaling pathway [87]. These findings reveal that an interaction between polyphenols and H_2_S might be behind the early-life origins of hypertension, although this remains speculative. 

Although several core molecular mechanisms were outlined above, additional work will need to be carried out to explore other potential mechanisms. A greater understanding of the interactions between individual polyphenols and the mechanisms implicated in their differential protective action will be the key to identifying proper implementation of polyphenols in early life for further clinical translation. 

## 6. Conclusions and Perspectives

Accumulating evidence in support of the beneficial role of early-life polyphenol supplementation in preventing hypertension of developmental origins is robust, but still incomplete. The biggest unsolved problem is the lack of a protective effect against programming of hypertension in humans by maternal dietary polyphenol consumption. Although more than 750 clinical trials have been performed on polyphenol-rich foods, polyphenol extracts, or their pure compounds to study their impact on health [133], presently, there is no information on how pregnant women receiving polyphenol supplementation will influence their children later in life. 

Another factor limiting the clinical translation of polyphenols is their low bioavailability in vivo [51]. In view of the complexity and inter-individual variability of polyphenol pharmacokinetics, additional research is needed to better explore the differential impact of various polyphenols on the early-life origins of hypertension. 

Another important aspect to consider is that substantial progress has been made in clarifying the benefits of different polyphenols in established hypertension, while little attention has been paid to their reprogramming effects in hypertension of developmental origins. In this review, only flavonols, flavanols, stilbenes, and tannins were investigated. Further examination will be required to get a fuller view of the reprogramming mechanisms of various polyphenols and test their dose-dependency using developmental programming models. 

In summary, polyphenols have a meaningful role in the prevention of hypertension. After gaining a better understanding of the mechanisms behind hypertension of developmental origins and the latest advances in the early use of polyphenols, further research in humans will be needed to provide important insights into clinical translation and reduce global hypertension rates.

## Figures and Tables

**Table 1:** Animal studies on polyphenols preventing hypertension in offspring.

Type and Dose	Animal Model	Species/ Gender	Age at Evaluation	Reprogramming Mechanisms	Ref.
**Flavonols**					
Quercetin (50 mg/kg/day) oral supplementation during gestation	Maternal high-fat diet	C57BL/6J mouse/M	6 months	Reduced oxidative stress	[85]
**Flavanols**					
Epigallocatechin gallate (458 mmol/L) in drinking water during gestation	Antenatal dexamethasone exposure	Wistar rat/M and F	14 weeks	Reduced oxidative stress	[86]
Garlic oil (100 mg/kg/day) oral gavage during gestation and lactation	Maternal high-fat diet	SD rat/M	16 weeks	Enhanced H_2_S generating enzymes, increased NO, altered gut microbiota	[87]
**Stilbenes**					
Resveratrol in drinking water (50 mg/L) during gestation and lactation	Maternal chronic kidney disease	SD rat/M	12 weeks	Reduced oxidative stress, restored NO, altered gut microbiota	[88]
Resveratrol in drinking water (50 mg/L) during gestation and lactation	Maternal and post-weaning high-fat diet	SD rat/M	16 weeks	Activated nutrient-sensing signals	[89]
Resveratrol in drinking water (50 mg/L) during gestation and lactation	Maternal L-NAME administration and high-fat diet	SD rat/M	16 weeks	Restored NO, activated nutrient-sensing signals, altered gut microbiota	[90]
Resveratrol in drinking water (50 mg/L) during gestation and lactation	Maternal ADMA and TMAO exposure	SD rat/M	12 weeks	Altered gut microbiota, blocked RAAS, restored NO	[91]
Resveratrol in drinking water (50 mg/L) during gestation and lactation	Maternal TCDD exposure	SD rat/M	12 weeks	Altered gut microbiota, antagonized AHR signaling, reduced renal inflammation	[92]
Resveratrol in drinking water (0.05%) during gestation and lactation	Maternal TCDD and dexamethasone exposure	SD rat/M	16 weeks	Reduced oxidative stress, restored NO, blocked the RAAS, and antagonized AHR signaling	[93]
Resveratrol in drinking water (50 mg/L) during gestation and lactation	Maternal bisphenol A exposure and high-fat diet	SD rat/M	16 weeks	Restored NO, reduced oxidative stress, antagonized AHR signaling	[94]
Resveratrol in drinking water (0.5%) at 2 to 4 months of age	Maternal and post-weaning high-fat diet	SD rat/M	16 weeks	Reduced oxidative stress, blocked RAAS, restored NO, activated nutrient-sensing signals	[95]
Resveratrol (4 g/kg of diet) during gestation and lactation	Maternal hypertension	SHR/M and F	20 weeks	Restored NO	[96]
**Tannins**					
Vitis vinifera L. grape skin extract (ACH09, 200mg/kg/day) during lactation	Maternal high-fat diet	SD rat/M	6 months	Reduced oxidative stress	[97]

Studies tabulated according to type of polyphenol, animal model, and age at evaluation. SD, Sprague–Dawley; SHR, spontaneously hypertensive rat; M, male; F, female; TCDD, 2,3,7,8-tetrachlorodibenzo-p-dioxin; L-NAME, N^G^-nitro-L-arginine-methyl ester; TMAO, trimethylamine N-oxide; NO, nitric oxide; RAAS, renin–angiotensin–aldosterone system; AHR, aryl hydrocarbon receptor.

## Data Availability

All data are contained within the article.
